# The Study of Ropivacaine Pharmacokinetics in a Clinical Setting: A Critical Scoping Review from the Perspective of Analytical Methodologies

**DOI:** 10.3390/ijms252413487

**Published:** 2024-12-16

**Authors:** Mihaela Butiulca, Lenard Farczadi, Camil Eugen Vari, Silvia Imre, Leonard Azamfirei, Alexandra Lazar

**Affiliations:** 1 Department of Anesthesiology and Intensive Care Medicine, Faculty of General Medicine, George Emil Palade University of Medicine, Pharmacy, Science, and Technology of Targu Mures, 540142 Târgu Mureș, Romania; mihaela.budrescu@umfst.ro (M.B.); leonard.azamfirei@gmail.com (L.A.); alexandralazar7@gmail.com (A.L.); 2 Department of Anesthesiology and Intensive Care Medicine, Emergency County Hospital, 540136 Târgu Mureș, Romania; 3 Chromatography and Mass Spectrometry Laboratory, Center for Advanced Medical and Pharmaceutical Research, George Emil Palade University of Medicine, Pharmacy, Science, and Technology of Targu Mures, 540142 Târgu Mureș, Romania; silvia.imre@umfst.ro; 4 Department of Pharmacology and Clinical Pharmacy, Faculty of Pharmacy, George Emil Palade University of Medicine, Pharmacy, Science, and Technology of Targu Mures, 540142 Târgu Mureș, Romania; camil.vari@yahoo.fr; 5 Department of Analytical Chemistry and Drug Analysis, Faculty of Pharmacy, George Emil Palade University of Medicine, Pharmacy, Science, and Technology of Targu Mures, 540142 Târgu Mureș, Romania

**Keywords:** ropivacaine, HPLC LC MS/MS, regional anesthesia

## Abstract

Ropivacaine, a widely used regional anesthetic also used for pain management, has been increasingly used in recent years due to its increased efficacy and improved safety compared to similar anesthetics. Biomonitoring of ropivacaine and its metabolites during and after anesthesia is an essential process for ensuring therapeutic efficacy and safe usage for patients. The most useful biomonitoring tool in recent years has been liquid chromatography coupled with mass spectrometry (LC-MS/MS), which offers selectivity, sensitivity, as well as accuracy of measurements. The current manuscript summarizes and discusses the existing liquid chromatographic methods described in the literature, as well as the personal experience with developing bioanalytical and analytical methods for the quantification of ropivacaine in biological samples for clinical applications. It is focused on methodological aspects, recent advancements, challenges, and future perspectives, highlighting the importance of LC-MS/MS techniques in ropivacaine analysis.

## 1. Introduction

### 1.1. History and Clinical Use of Ropivacaine

Pain, an unpleasant sensory and emotional experience linked to actual or potential harm, can be managed through various techniques and medications to ensure patient comfort [1]. Among the most used local anesthetics are lidocaine, articaine (esters), ropivacaine, and bupivacaine (amides). Recently, ropivacaine has gained popularity due to its extended effect and superior safety profile compared to bupivacaine.

Ropivacaine was first synthesized in the late 1970s and early 1980s to create safer alternatives to established local anesthetics such as bupivacaine. Initial investigations concentrated on understanding its pharmacological characteristics and evaluating its potential for clinical application [2].

### 1.2. Pharmaceutical Formulation and Administration

Ropivacaine is commercially available in multiple concentrations, including 0.2%, 0.4%, 0.7%, and 1%, with the selection depending on the specific procedure and the required duration of anesthesia. It is widely utilized in various nerve block techniques, such as epidural anesthesia for labor and delivery, peripheral nerve blocks in orthopedic surgeries, and local infiltration anesthesia for minor procedures [3]. To enhance its effects, ropivacaine can be combined with various adjuvants, among the most notable being epinephrine, dexamethasone, dexmedetomidine, and magnesium [4].

### 1.3. Indications and Contraindications

Ropivacaine is primarily used for surgical anesthesia, labor pain, and postoperative pain management in adults and children. It can be administered epidurally (commonly for cesarean sections and hip or lower limb surgeries, but also following abdominal surgeries), intrathecally, or via peripheral nerve blocks for anesthesia or post-surgery pain management. Ropivacaine can also be beneficial for patients with chronic or malignant pain. It is shown to be more effective in pain relief as a local analgesic than intravenous morphine, with a lower risk of complications than general anesthesia [5].

Ropivacaine is contraindicated in individuals allergic to local anesthetic agents, those with sepsis, regional infections, and unstable hemodynamics, and it should not be used for Bier’s block and paracervical block [6].

### 1.4. Special Situations

Ropivacaine has demonstrated anti-proliferative and pro-apoptotic effects in various cancers, including hepatocellular and malignant melanoma [7,8]. Another significant characteristic of ropivacaine is its in vitro antibacterial activity, which inhibits the growth of *Staphylococcus aureus*, *Escherichia coli*, and *Pseudomonas aeruginosa* [9,10].

### 1.5. Adverse Effects

Ropivacaine’s toxicity manifests as CNS events, including nervousness, tingling around the mouth, tinnitus, tremor, dizziness, blurred vision, and seizures, followed by respiratory depression, apnea, loss of consciousness, and cardiovascular effects such as hypotension, bradycardia, arrhythmias, and cardiac arrest [11]. Adverse effects vary depending on the individual profile of each patient. In some cases, a brief prodromal phase precedes the onset of severe adverse effects, whereas in others, adverse effects may manifest abruptly such as convulsive seizures.

### 1.6. Risk Factors

The American Society of Regional Anesthesia and Pain Therapy (ASRA) has classified the risk factors for systemic toxicity caused by local anesthetics into patient, anesthetic, and practice setting-related risk factors [11]. Patient age is a significant aspect of local anesthetic systemic toxicity (LAST), with the extremes of age often implicated in the negative effects of a procedure [12]. Other factors, such as hypoalbuminemia, low muscle mass in geriatric patients, and liver and kidney impairment, increase the incidence of narcotic events [13]. Due to physiological changes during pregnancy, patients in this situation are more likely to develop LAST [14].

The choice of anesthetic technique significantly influences the occurrence of Local Anesthetic Systemic Toxicity (LAST). Studies indicate that penile and caudal blocks are often associated with LAST, likely due to their common use in infants. Additionally, prolonged continuous administration of ropivacaine has been linked to toxicity cases [15].

As the saying goes, “Prevention is better than cure”. The primary objective should be to prevent and circumvent LAST in regional anesthesia practice. This can be achieved through multiple safety measures, vigilant monitoring, and avoidance of risk factors, all aimed at preventing or mitigating the severity of LAST [15].

### 1.7. LAST Management

Weinberg’s review outlines the recommended management strategies for LAST. According to various studies, rapid airway management is crucial to maintain the acid-base balance and prevent severe complications [16,17]. This can be accomplished through advanced ventilation techniques or airway prosthetics. Another key aspect of toxicity management is the suppression of seizures, which plays a significant role in preventing metabolic acidosis. The cautious administration of propofol and benzodiazepines is advised [18]. If toxicity is not correctly assessed and addressed, it can quickly escalate into cardiovascular instability, necessitating careful management to maintain coronary perfusion and prevent catastrophic cardiovascular arrest. The use of antiarrhythmic medication, vasopressors if needed, and mechanical devices to sustain vital functions is recommended. Since 2001, lipid infusion has been widely used to treat local anesthetic toxicity [19].

### 1.8. Pharmacological and Chemical Properties

**Figure 1:**
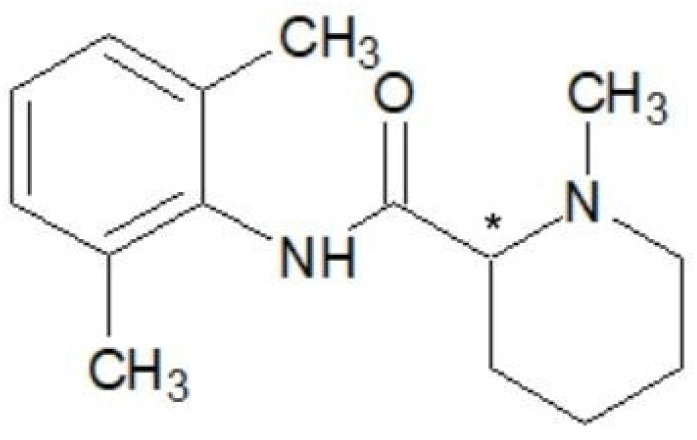
Chemical structure of mepivacaine. Chiral carbon atom is marked with asterisk.

**Figure 2:**
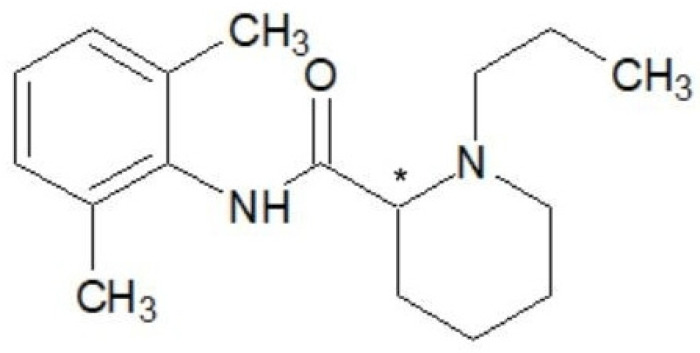
Chemical structure of ropivacaine. Chiral carbon atom is marked with asterisk.

**Figure 3:**
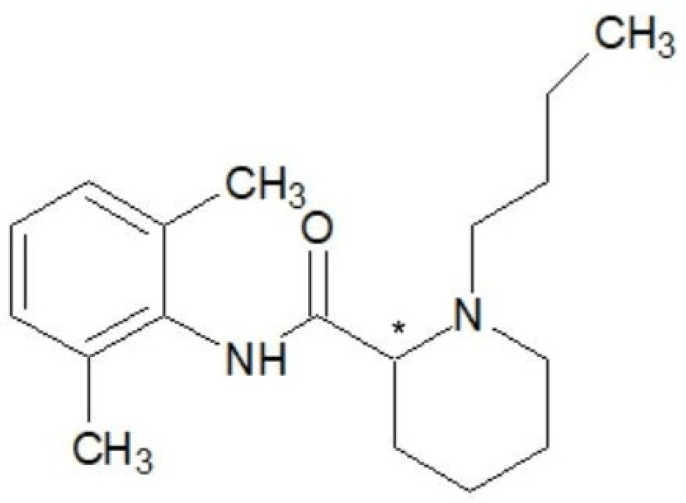
Chemical structure of bupivacaine. Chiral carbon atom is marked with asterisk.

Ropivacaine, the first long-acting local anesthetic of pure S chirality, is known chemically as S-1-propyl-2,6-pipecoloxylide hydrochloride monohydrate. It has a molecular weight of 274 kDa, a pKa of 8.07, and a pH of 7.4 [20]. It shares a similar chemical structure with bupivacaine and mepivacaine, with the only difference being the modification of the groups attached to the nitrogen atom [21]. For instance, compared to mepivacaine, which has a methyl(-CH3) group attached to the nitrogen atom, ropivacaine has a propyl(-C3H7) group, and bupivacaine has a butyl(-C4H9) group. The lipophilicity of these compounds, which influences the potency of anesthetics, varies according to the length of the carbon chain [22] (Figure 1, Figure 2 and Figure 3).

Enantiomers exist in two spatial configurations and are present in equal amounts in a racemic mixture. Although they are physiochemically similar, the two structures behave differently depending on their affinity for a certain type of action. Typically, the S- enantiomer has an affinity for the desired effects of the drug, while the R+ enantiomer may have a higher affinity for the undesired effects. The increased cardiotoxicity associated with the R-configuration could be due to the stereo-selective uptake of local anesthetics by cardiac sodium channels [23].

Thanks to advancements in extraction methods and stereo-selective synthesis, scientists have been able to commercially produce local anesthetics as a single enantiomer. This led to the creation of ropivacaine, which at the time of its marketing, consisted of more than 99% S-form. It is a pure S(−) enantiomer, developed as a safer anesthetic to reduce cardiac and neurological toxicity. In animals and healthy individuals, ropivacaine has a significantly higher threshold for cardiotoxicity and Central Nervous System toxicity than bupivacaine due to its lower lipophilicity and stereoselective characteristics [24].

### 1.9. Pharmacodynamics

Ropivacaine works by reversibly suppressing the influx of sodium ions, thereby preventing impulse conduction in nerve fibers. It also has a dose-dependent inhibitory effect on potassium ion channels. Its selective impact on pain transition (A and C nerves) over large, myelinated motor fibers (A fibers) can be attributed to its reduced lipophilicity compared to bupivacaine [25].

### 1.10. Pharmacokinetics

The systemic absorption rate of ropivacaine determines plasma concentrations and the time required to reach the desired peak. High plasma concentrations are associated with systemic toxicity. However, the maximum doses required for the administration of this substance are not clearly understood, especially considering the comorbidities of patients who receive this type of local anesthetic. Local toxicity has been observed in patients who received doses within the recommended reference range, as well as in patients who received higher doses without suffering from the effects of LAST. The pharmacokinetics of ropivacaine are highly dependent on the administered doses, the route of administration, and the patient’s comorbidities [26,27].

### 1.11. Volume of Distribution

Ropivacaine, after intravenous infusion, has a distribution volume of 41 ± 7 L, a plasma clearance of 387 ± 107 mL/min, and a renal clearance of 1 mL/min. It has a half-life of 1.8 ± 0.7 h post-intravascular administration and 4.2 ± 1-h post-epidural administration. Notably, ropivacaine can cross the blood–placental barrier [28].

Ropivacaine’s primary metabolism is facilitated by cytochrome P450 (CYP) 1A2, leading to the formation of 3-OH-ropivacaine through aromatic hydroxylation, and by the CYP3A4 enzyme, resulting in the metabolite 2′, 6′-pipecoloxylidide through N-dealkylation. Ropivacaine also produces two minor metabolites: 4-OH-ropivacaine and 2-OH-methyl ropivacaine. About 94% of the substance binds to plasma proteins, particularly 1-acid glycoprotein. The plasma concentration of the substance is determined by the total dose administered, the administration method, the patient’s hemodynamic status, and the vascularity of the infiltration site. The kidney, as the primary excretory organ for ropivacaine, accounts for 86% of the drug’s excretion following a single intravenous dose administration [29].

### 1.12. Biomonitoring and Pharmacokinetics

Biomonitoring studies are an increasingly important aspect of the clinical monitoring of patients after administration of different types of drugs. They give important information on the particularities of each patient and how their body processes drug molecules. By measuring and monitoring drug levels after administration, not only can physicians adjust treatment and improve effectiveness, but can also reduce adverse effects or detect certain metabolic particularities for everyone [30].

Pharmacokinetics refers to the use of mathematical models to describe and predict drug concentrations in organs and/or body fluids over time. While there are various ways to predict what a living organism does to an active pharmaceutical ingredient, such as non-compartmental analysis, compartmental analysis, and physiologically based pharmacokinetic analysis, pharmacokinetic studies always require several bioanalytical measurements on which to base the mathematical model and the predictions. As such, based on a limited number of scientific measurements, pharmacokinetic analysis can be used to obtain a set of parameters that describe the entire kinetics of the drug molecule after administration in the body. Pharmacokinetic evaluation is used in different types of bioavailability studies to extrapolate results obtained on animal-model or human studies and obtain a complete image of how a drug is distributed, metabolized, and eliminated from the body [31].

### 1.13. Bioanalytical Techniques and Methodologies

Simple bioanalytical techniques that yield reliable results are very important in real-time clinical biomonitoring as they make quick and precise diagnosis possible and enable adjustment for appropriate treatment. This, in turn, is essential for modern, patient-focused, and personalized treatments, improving the correct and proper dosage of active pharmaceutical ingredients on a case-by-case basis and lowering the risk of complications and adverse effects. In pharmacokinetic studies, obtaining accurate and reliable results can give valuable information on short- and long-term effects that can occur after administration.

There are many bioanalytical techniques that are generally used for biomonitoring and pharmacokinetics studies and which can be applied when studying ropivacaine and its metabolites. The most widely used techniques involve liquid chromatography (HPLC) for analyte separation coupled with different detectors for analyte detection, such as UV detectors (HPLC-UV) or fluorescence detectors (FLD), but also with mass spectrometric detection (LC-MS). Most often, ropivacaine and its metabolites are measured and monitored in biological matrices such as urine and blood. Methods such as gas chromatography (GC), sometimes also coupled with mass spectrometric detection (GC-MS), or enzyme-linked immunosorbent assay (ELISA) can also be used in biomonitoring, pharmacokinetic, and bioavailability studies but are not suitable for any type of compounds.

When choosing a technique and developing a suitable method one must consider not only the details of the application and the scope of the measurements but also, regardless of the technique chosen, the method must be optimized for proper sensitivity, selectivity, and robustness to assure accurate, precise, and reliable results. Although not always thought of as an influencing factor, the equipment available and its performance are also critical. They not only influence the cost of the analytical determination but can be a decisive factor in the feasibility of a method and/or project. Each technique has advantages and disadvantages. Some might have better sensitivity, selectivity, etc., but for certain applications, simpler and less high-performing techniques might be sufficient. They can offer a more cost-effective alternative, simpler sample cleanup due to the reduced sensitivity and thus less time consumption, or various other advantages [32].

## 2. Methods

This review was reported according to the Preferred Reporting Items for Systematic Review and Meta-analysis (PRISMA) Extension for Scoping Reviews (PRISMA-ScR) checklist. The review protocol was registered with Open Science Framework (Registration DOI: https://doi.org/10.17605/OSF.IO/28X4M). The research question for this study was “What types of methods are described in scientific literature and what is their applicability in the study of ropivacaine pharmacokinetics in a clinical setting?”.

We propose an integrated and synthesized examination of the present knowledge concerning liquid chromatographic methods and strategies for biomonitoring ropivacaine and other local anesthetics used in clinical practice. Our discussion encompasses various approaches, their outcomes, and the advantages and disadvantages of commonly employed techniques. We endeavor to highlight key considerations in method development for identifying and quantifying local anesthetics tailored to specific applications.

**Figure 4:**
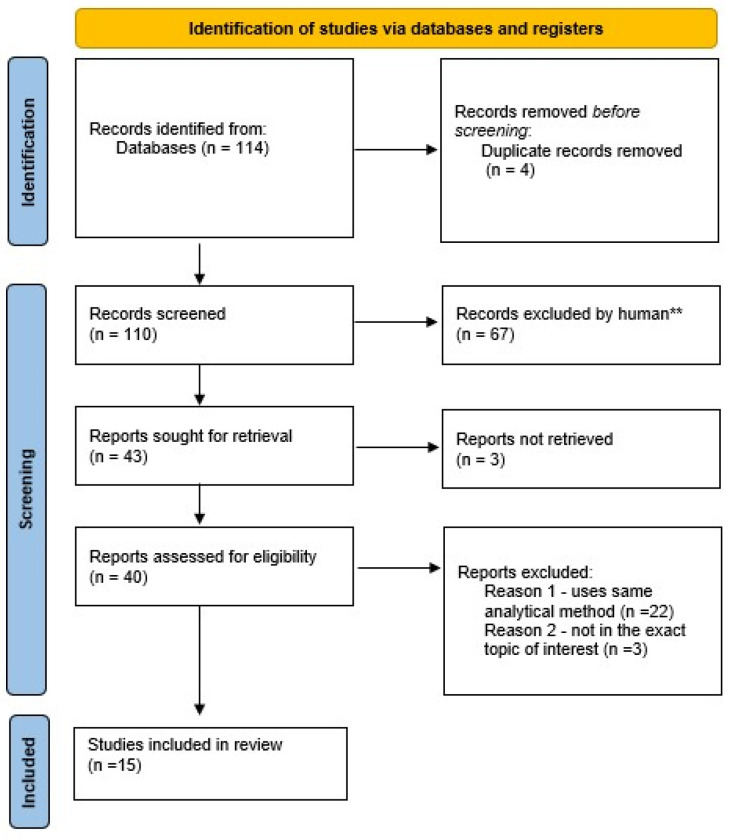
PRISMA flow diagram of databases research [33] (** processed by human).

In this review, we selected the most pertinent publications from the scientific literature of the past 12 years, utilizing renowned databases such as PubMed and Web of Science. Our search, conducted with terms like ‘ropivacaine’, ‘ropivacaine quantification’, ‘ropivacaine biomonitoring’, and ‘local anesthetics biomonitoring’, ‘local anesthetics quantification’, ‘ropivacaine LC-MS/MS’ was last updated in September 2024. Notably, we did not assess the quality or rank of the publications (Figure 4) [33].

Our analysis focused on research papers detailing liquid chromatographic methods for detecting ropivacaine in human biological samples. Primarily, we address the challenges associated with selecting and refining appropriate analytical methods for biomonitoring in clinical practice (Table 1).

By amalgamating these approaches, our objective was to identify inconsistencies in previous findings, elucidate the strengths and weaknesses of diverse methods, and pinpoint existing gaps to present as much as possible a wide perspective for those who are interested in starting their own chromatographic method development for the determination of ropivacaine concentrations in human fluids. To accomplish this, in the present article, a descriptive analysis was carried out by taking into consideration key aspects of LC methodologies, underlining sample preparation and range of concentration for a rapid orientation according to the aim of future bioanalytical studies.

## 3. Results and Discussion

While ropivacaine biomonitoring can be important on its own due to certain unavoidable risks concerning general anesthesia, this is even more important in certain cases, such as novel types and methods of anesthesia procedures, necessity of prolonged anesthesia, or certain comorbidities or health risks that increase the risk of adverse drug effects or toxicity. Biomonitoring or bioanalytical measurement is also essential in certain types of studies, such as bioavailability studies (for example, after novel anesthetic procedures or surgical procedures), drug interactions studies (for the medication of patients, or for interactions with illegal substances), or toxicity and forensics studies. Several types of quantification methods are described in the literature, using different types of bioanalytical techniques. While most methodologies described use liquid chromatography for analyte separation (HPLC, LC-MS/MS), due to the physical and chemical properties of ropivacaine and its metabolites, there are few methods that use different separation techniques, such as gas chromatography (GC), which are sometimes also coupled with mass spectrometric detection (GC-MS). While there is no straightforward answer to which methodology and technique are the most suitable, it depends on several factors, including but not limited to equipment availability, expertise and experience of personnel, study design, and expected quantification limit, as well as the cost-to-benefit ratio. While some techniques might indeed offer better performance with regards to selectivity and sensitivity (for example, mass spectrometric detection), if the sensitivity granted by other detectors (such as UV) is deemed sufficient, it might be used due to the generally better robustness of UV detection compared to MS. At the same time, however, due to reduced selectivity, more cumbersome and time-consuming sample cleanup and preparation might need to be used to eliminate possibly interfering compounds from the analyzed samples. Similarly, the better selectivity of MS detection can, in certain cases, offer a reduced need for chromatographic separation, as a pseudo-separation can be achieved by extracting specific mass chromatograms from the total ion chromatogram, thus allowing for shorter runtimes, easier analysis of multiple analytes (such as metabolites and comedication) for biomonitoring purposes, and sometimes can even allow for shorter method development times due to this superior selectivity. The last aspect to mention is the difference in cost between HPLC-UV and LC-MS/MS, with both equipment acquisition and maintenance, cost of consumables, parts, and reagents (which need to be of higher purity for LC-MS to avoid contaminations), service costs in case of malfunctions, but also costs of training personnel being higher for the more advanced LC-MS/MS technique. Basically, the choice is down to a case-by-case basis. Even though LC-MS/MS might seem the more modern and advantageous methodology, in certain cases, HPLC-UV can suffice or can offer a better cost-to-benefit ratio under certain circumstances. We aimed to describe and summarize the methods used and described in the literature to help analysts and healthcare specialists get a clear overview of the available possibilities for ropivacaine quantification and biomonitoring (Table 2).

Rifai et al. [34] described one of the earlier techniques of ropivacaine and bupivacaine quantification from plasma, using reversed-phase liquid chromatography with UV detection. Reversed-phase chromatography is the most used HPLC separation technique. It is used for the separation of compounds that have hydrophobic fragments and do not have a dominant polar character. The method uses pentycaine as an internal standard and chromatographic separation is performed using isocratic elution with a mobile-phase mixture of acetonitrile, methanol, and phosphate buffer solution (pH 6.0) (volume ratio 1:1:3) and a 6 min runtime for the separation and simultaneous determination of both ropivacaine and bupivacaine. The column used was an LC8 DB column (5 μm particle size, 5.0 cm × 4.6 mm). The calibration range was between 200 and 1000 ng/mL, and samples were prepared using solid-phase extraction.

The method developed by Yu et al. [35] for ropivacaine and bupivacaine quantification from plasma, another early study on these molecules, also uses the HPLC-UV technique. Pentrycaine was the internal standard and samples were purified by online solid-phase extraction on a HPLC system capable of column switching. The analytical separation was performed on a Hypercarb analytical column (size 100 × 3.2 mm) and mobile phase consisting of acetic acid, triethylamine, methanol, isopropanol in 0.01 M N-(2-acetamido)-2-amino-ethanesulfonic acid buffer, and ammonia solution in isocratic elution. The calibration range of the method was between 1.6 and 120 μg/mL, which is appropriate for the technique used, especially at the time of publication.

Another HPLC-UV method, described by Tanaka et al. [36], was developed for the determination of three local anesthetic drugs from the pipecoloxylidide group in human serum (mepivacaine, bupivacaine, and ropivacaine). Tetracaine was used as an internal standard. Serum standards for calibration were prepared at 2, 10, 50, 100, 200, 500, and 1000 ng/mL, giving the method a wide calibration range. Analytes were separated on an Inertsil C18 reversed-phase column (column dimensions 150 × 4.6 mm, particle size of 5 μm) using a mobile-phase mixture of acetonitrile, methanol, and monosodium phosphate in isocratic elution. Analytes were detected at the wavelength of 210 nm. The sample cleanup was performed using protein precipitation, solvent evaporation, and, finally, resolubilization of analytes in an appropriate solvent.

Gaudreault et al. [37] developed a method for the simultaneous quantification of ropivacaine and bupivacaine, using tetracaine as the internal standard, which also uses the HPLC-UV technique. Analyte separation was performed on a C8 reversed-phase column (size 150 × 4.6 mm, particle diameter 5 μm) and mobile phase consisting of a mixture of sodium sulfate and acetonitrile (7:3, *v*/*v*) in isocratic elution, with detection being performed at 205 nm. The calibration range for ropivacaine was between 4 and 1000 ng/mL. Samples were purified using a complex sample preparation method involving multiple steps, including liquid–liquid extraction.

Mouzi et al. [38] quantified ropivacaine in plasma from subjects undergoing unilateral total knee replacement or anterioar cruciate ligament reconstruction under general or spinal anesthesia. They used a HPLC-UV (detection at 205 nm) method, bupivacaine being the internal standard, with analytical separation being performed on a C18-type reversed-phase column and mobile phase consisting of 10 mM potassium dihydrogen phosphate and acetonitrile 73:27 (*v*/*v*) in isocratic elution. Plasma samples were analyzed following extraction in ethyl ether from the alkalized sample, re-extraction from the organic phase in an acidic solution, and, finally, were diluted with a sodium acetate solution. The calibration range for ropivacaine was extended to a greater upper limit of quantification than previous described papers and ranged between 50 and 5000 ng/mL.

Kawata et al. [39] developed an assay method for the determination of plasma ropivacaine by using HPLC-UV. Analytical separation was performed using a mobile phase composed of acetonitrile, methanol, and 0.05 M phosphate buffer adjusted to pH 4.0 in isocratic elution (at a flow rate of 0.8 mL/min) and a C18 TSK-GEL column with a 4.6 × 150 mm size. Analyte detection was performed in a UV domain at 215 nm. The method uses bupivacaine as an internal standard, and liquid–liquid extraction for sample clean-up. The calibration range of ropivacaine was between 25 and 1000 ng/mL.

During this time, mass spectrometry detection was also involved for LC determination of ropivacaine in humans, dealing with the lower limit of quantification and by high throughput sample preparation.

Zhang et al. [40] describe a technique of quantification ropivacaine from plasma using LC-MS/MS. Sample preparation used equilibrium dialysis, a technique by which only free plasma ropivacaine concentrations can be measured, but not protein-bound ropivacaine. This technique is complex but very limited in scope and usability.

Koehler et al. [41] also described a different technique of detection using LC-MS/MS for the quantification of bupivacaine, mepivacaine, prilocaine, and ropivacaine from human serum. No internal standard was used for this method. The chromatographic separation was performed on a Synergy 4 μm Polar-RP column (150 × 2 mm) with a mobile-phase mixture of acetonitrile, 2 mM ammonium acetate and formic acid, a gradient elution, and a total run-time of 7 min for each sample. Biological samples were prepared using a simple protein precipitation method. The use of mass spectrometric detection allows for a lower calibration range of concentrations between 1 and 200 ng/mL of ropivacaine.

Mathiue et al. [42] also developed a method using the same LC-MS/MS type of technique for the quantification of ropivacaine. They used an XD8 C8 column (5 μm particles, dimensions of 150 × 4.6 mm) for analyte separation with a mobile-phase mixture of trimethylamine in acetonitrile and ammonium formate buffer and a 15 min gradient elution. The internal standard used was etidocaine, and samples were purified by equilibrium dialysis to measure free plasma ropivacaine and protein precipitation for total plasma ropivacaine concentration measurement.

Sawaki et al. [43] developed a LC-MS/MS method that uses 10 mM ammonium acetate and acetonitrile in isocratic elution on a long Chemcosorb 7-ODS-H (250 × 4.6 mm) analytical column. Plasma samples were precipitated using 30% trichloroacetic acid, which dilutes the sample very little and allows for higher sensitivity. Also, as the protein precipitation method was used, which has a high recovery rate, the authors decided that the method needs no internal standard.

Breindahl et al. [44] developed a method based on a LC system in tandem with a QTRAP mass spectrometer by using a deuterated internal standard to improve robustness (ropivacaine-D7). This method uses a short Agilent LC column.

Another chromatographic method for the quantification of ropivacaine and its main active metabolite (3-OH-ropivacaine) using LC-MS/MS was developed by Butiulca et al. [48] and was successfully applied in several human studies where the bioavailability and metabolic pathways of ropivacaine were analyzed after plane block anesthesia. The method used reversed-phase chromatographic separation of analytes on a C18-type column and a 7 min mobile-phase gradient of two liquid phases: acetonitrile and 0.05% aqueous formic acid. The short runtime was coupled with a simple and quick protein precipitation for sample cleanup, thus allowing for high-throughput analysis for biomonitoring purposes. The calibration intervals of this method were 0.5–1000 ng/mL for ropivacaine and 1–1000 ng/mL for 3-OH-ropivacaine, and the method was validated to be robust, accurate, and linear over these calibration ranges.

Important aspects to take into consideration when choosing an analytical or bioanalytical method for sample analysis can be both economical in nature (cost of analysis reagents and consumables, complexity and know-how needed from personnel, cost of equipment acquisition and maintenance) as well as practical (performance needed, equipment available, time constraints, sample quantity available, etc.), but in certain cases, other considerations might be more important, such as, for example, the use of less toxic substances to increase analyst safety, the use of less polluting and safer reagents to reduce environmental pollution, or the novelty factor of the methodology. As such, depending on the priorities, a case-by-case analysis must be made in order to choose the most suitable method from the wide array of the available scientific literature.

While some described methods use simpler and generally more inexpensive analytical techniques (such as HPLC-UV), the downside is that there is a need to purify samples more rigorously before this type of analysis, as the selectivity of UV detection is lower compared to mass spectrometric detection. Mass spectrometric detection (used in LC-MS/MS, especially in the case of ropivacaine) has the advantage of needing minimal sample purification due to the very high selectivity and sensitivity. Usually, a simple protein precipitation will suffice; however, the costs of acquiring and maintaining such equipment are much higher compared to UV detectors. The trend, however, is clear: most methods described in the literature use LC-MS/MS as the analysis technique due to the uncompromised reliability of measured results despite the somewhat higher costs of operation and the need for higher qualification of personnel.

## 4. Conclusions

While there is no definitively true answer to which method is the best when it comes to ropivacaine biomonitoring and pharmacokinetics studies, as there are advantages and disadvantages to every analysis technique, we presented a short overview of currently described methodologies. The answer to which analytical method fits best must be studied by each analyst and researcher individually, on a case-by-case basis, and decisions must be taken based on the availability of equipment and know-how in the laboratory, as well as depending on the availability of funding, deadlines, and other, more subjective factors (for example, the need of a perceived impressiveness some methods offer). The LC methods summarized in this research paper offer a wide variety of options, each with its own advantages and drawbacks, some only slight, some more significant, but in the end, the final decision on which analytical methodology should be used has to be taken by each research group based on their needs. While this manuscript was envisioned as a comprehensive scoping review at the time of writing, one must consider that the field of bioanalytical methodologies is ever developing, and there is no definitive “best method” when it comes to a field as varied as clinical and biomedical research bioanalytical chemistry.

## Figures and Tables

**Table 1:** Advantages and disadvantages of analytical methods.

Method	Advantages	Disadvantages
HPLC-UV	high reproducibility, simpler sample preparation, lower cost, widely available	low selectivity, low sensitivity
(HP)LC-MS	high selectivity, high sensitivity, simpler sample preparation	lower reproducibility, higher cost, not widely available
HPLC-FLD	higher specificity	not widely available, complicated/costly sample preparation (derivatization)
GC(-MS)	higher specificity, higher sensitivity, widely available	complicated/costly sample preparation (derivatization)
ELISA	simple sample preparation, low cost	equipment widely available but kit not always available

**Table 2:** Selected methods described in the scientific literature for biomonitoring ropivacaine and other local anesthetics exposure.

Biological Matrix	Column	Analytical Separation and Detection Method	Sample Preparation Technique	Biomarker of Exposure	Internal Standard	Calibration Range	Author
plasma	LC8 DB column (5 μm particle size, 5.0 cm × 4.6 mm)	HPLC-UV	solid-phase extraction	ropivacaine, bupivacaine	pentycaine	200–1000 ng/mL	Rifai et al. [34]
plasma	Hypercarb analytical column (size 100 × 3.2 mm)	HPLC-UV	solid-phase extraction	ropivacaine, bupivacaine	pentycaine	1.6–120 μg/mL	Yu et al. [35]
human serum	Inertsil C18 reversed-phase column (150 mm × 4.6 mm, particle and 5 μm)	HPLC-UV	protein precipitation, solvent evaporation, resolubilization	mepivacaine, bupivacaine, ropivacaine	tetracaine	2–1000 ng/mL	Tanaka et al. [36]
plasma	C8 reversed-phase column (size 150 × 4.6 mm, particle diameter 5 μm)	HPLC-UV	Liquid–liquid extraction	ropivacaine, bupivacaine	tetracaine	4–1000 ng/mL	Gaudreault et al. [37]
plasma	C18 type reversed-phase column	HPLC-UV	Ethylether extraction, solvent evaporation	ropivacaine	bupivacaine	50–5000 ng/mL	Mouzi et al. [38]
plasma	C18 column TSK-GEL with 4.6 × 150 mm size	HPLC-UV	Liquid–liquid extraction	ropivacaine	bupivacaine	25–1000 ng/mL	Kawata et al. [39]
plasma	not specified	not specified	equilibrium dialysis	ropivacaine	not specified	not specified	Zhang et al. [40]
human serum	Synergy 4 μm Polar-RP column (150 mm × 2 mm)	LC-MS/MS	protein precipitation	bupivacaine, mepivacaine, prilocaine, ropivacaine	no internal standard	1–200 ng/mL	Koehler et al. [41]
plasma	XD8 C8 column (5 μm particles, dimensions of 150 × 4.6 mm)	LC-MS/MS-ESI	protein precipitation	ropivacaine	etidocaine	50–400 μg/L	Mathieu et al. [42]
plasma	Chemcosorb 7-ODS-H (250 × 4.6 mm)	LC-MS/MS	protein precipitation	ropivacaine	no internal standard	not specified	Sawaki et al. [43]
plasma, ultrafiltrate, drainage blood	Agilent Zorbax SB-Aq column (50 × 2.1 mm)	LC-MS/MS	sample dilution, protein precipitation, and ultrafiltration	ropivacaine	ropivacaine-D7	0.1–10 µg/mL	Breindahl et al. [44]
human serum	Mightysil-RP-18 GP II column (150 × 2 mm, particle size 5 μm)	LC-MS/MS-ESI	solid-phase extraction	procaine, mepivacaine, lidocaine, ropivacaine, oxybuprocaine, tetracaine, bupivacaine, T-caine and dibucaine	lidocaine	10–100 ng/mL	Tonooka et al. [45]
human serum	reversed-phase column	LC-MS/MS-ESI	equilibrium dialysis	free and total ropivacaine	ropivacaine-D7	0.5–3000 ng/mL	Lamy et al. [46]
human serum	Optiguard C8 column (1 × 10 mm)	LC-MS/MS-ESI	solid-phase microextraction	ropivacaine, PPx, 3-OH-ropivacaine	ropivacaine-D7	2–2000 nM for all analytes	Abdel-Rehim et al. [47]
human serum	Gemini NX-18 (3 × 100 mm)	LC-MS/MS-ESI	protein precipitation	ropivacaine, 3-OH-ropivacaine	ropivacaine-D7	0.5–1000 ng/mL for ropivacaine, 1–1000 ng/mL for 3-OH-ropivacaine	Butiulca et al. [48]

## Data Availability

No new data were created or analyzed in this study.
